# Short-term forecasting of the prevalence of clinical trachoma: utility of including delayed recovery and tests for infection

**DOI:** 10.1186/s13071-015-1115-8

**Published:** 2015-10-22

**Authors:** Fengchen Liu, Travis C. Porco, Abdou Amza, Boubacar Kadri, Baido Nassirou, Sheila K. West, Robin L. Bailey, Jeremy D. Keenan, Thomas M. Lietman

**Affiliations:** F.I. Proctor Foundation, University of California San Francisco, 513 Parnassus, Medical Sciences 309A, San Francisco, CA 94143-0944 USA; Department of Ophthalmology, University of California San Francisco, San Francisco, CA USA; Department of Epidemiology and Biostatistics, University of California San Francisco, San Francisco, CA USA; Programme FSS/Université Abdou Moumouni de Niamey, Programme National de Santé Oculaire, Niamey, Niger; Dana Center for Preventive Ophthalmology, Wilmer Eye Institute, Johns Hopkins University, Baltimore, MD USA; Clinical Research Unit, Department of Infectious and Tropical Diseases, London School of Hygiene and Tropical Medicine, London, UK

**Keywords:** Trachoma, Model, Mass drug administration, Forecast, Prediction

## Abstract

**Background:**

The World Health Organization aims to control blinding trachoma by 2020. Decisions on whether to start and stop mass treatments and when to declare that control has been achieved are currently based on clinical examination data generated in population-based surveys. Thresholds are based on the district-level prevalence of trachomatous inflammation–follicular (TF) in children aged 1–9 years. Forecasts of which districts may and may not meet TF control goals by the 2020 target date could affect resource allocation in the next few years.

**Methods:**

We constructed a hidden Markov model fit to the prevalence of two clinical signs of trachoma and PCR data in 24 communities from the recent PRET-Niger trial. The prevalence of TF in children in each community at 36 months was forecast given data from earlier time points. Forecasts were scored by the likelihood of the observed results. We assessed whether use of TF with additional TI and PCR data rather than just the use of TF alone improves forecasts, and separately whether incorporating a delay in TF recovery is beneficial.

**Results:**

Including TI and PCR data did not significantly improve forecasts of TF. Forecasts of TF prevalence at 36 months by the model with the delay in TF recovery were significantly better than forecasts by the model without the delay in TF recovery (*p* = 0.003). A zero-inflated truncated normal observation model was better than a truncated normal observation model, and better than a sensitivity-specificity observation model.

**Conclusion:**

The results in this study suggest that future studies could consider using just TF data for forecasting, and should include a delay in TF recovery.

**Trial registration:**

Clinicaltrials.gov NCT00792922

**Electronic supplementary material:**

The online version of this article (doi:10.1186/s13071-015-1115-8) contains supplementary material, which is available to authorized users.

## Background

The World Health Organization (WHO), the International Trachoma Initiative (ITI), Ministries of Health, and their partners aim to control blinding trachoma by 2020 by implementing surgical campaigns, antibiotic distributions, hygiene initiatives, and environmental improvements [1, 2]. Decisions on whether to start and stop mass treatments and when to declare that control has been achieved are currently based on the clinical examination data generated in population-based surveys. Thresholds are based on the district-level prevalence of trachomatous inflammation–follicular (TF) in children aged 1–9 years. Forecasts of which districts may and may not meet TF control goals by the 2020 target date could affect resource allocation in the next few years.

Unfortunately, TF is not an ideal indicator for several reasons. TF is only indirectly associated with infection with the causative agent *Chlamydia trachomatis*, in part because the clinical signs of trachoma may persist for months after infection has been cleared [3–7]. Also, TF is a subjective sign with only moderate reproducibility. Other indicators such as trachomatous inflammation-intense (TI) and more direct evidence of chlamydial infection (PCR) may provide additional information, even in forecasting the future prevalence of TF.

Recent community-randomized trials have followed multiple communities which were treated with identical mass antibiotic programs, providing datasets to test various types of forecasts. These trials have assessed the prevalence of TF, TI and chlamydial PCR. Here, we use a hidden Markov model with recent clinical trial data of biannual assessments of 24 communities from baseline through 30 months to forecast the prevalence of TF in children in each at 36 months [8]. We assess whether use of TF with additional TI and PCR data rather than just the use of TF improves forecasts, and separately whether incorporating a delay in TF recovery is beneficial; we also compare different observation models.

## Methods

### Data collection

Forty-eight communities were followed as part of the Niger arm of the Partnership for the Rapid Elimination of Trachoma (PRET) study. Communities were randomized to either mass antibiotics of the entire community, or antibiotics targeted just to children 12 years and younger. The 24 communities included in this study received annual antibiotic treatment of all ages. Communities were assessed at baseline and then biannually for 3 years. All individuals were offered antibiotic treatment annually, within two weeks of the assessment: children under 6 months, those allergic to macrolides, and pregnant women were offered topical tetracycline, and all others were offered a single dose of oral azithromycin (20 mg/kg for children and 1 g for adults).

A random sample of 100 children 0–5 years old were selected from each community. If a community had less than 100 0–5 year-old children, then all were offered assessment. Each participating child had their upper right tarsal conjunctiva swabbed, and processed for PCR as previously described [9]. Clinical grading of the right everted superior tarsal conjunctiva was performed using a 2.5× magnifying loupe and adequate sunlight or a torch light according to the WHO simplified grading system [10] as previously described [9].

### Ethics statement

This study of de-identified data received ethical approval from the Committee on Human Research of the University of California San Francisco and was carried out in accordance with the Declaration of Helsinki. A parent or guardian of any child participant provided informed consent on their behalf. The informed consent given was oral: (a) we chose verbal consent because of the low literacy rates in the study area, (b) the IRB (10.00812) approved the use of oral consent, and (c) oral consent was documented on the registration form for each study participant prior to examination in the field.

### Modeling methods

We constructed a stochastic transmission model of *Chlamydia trachomatis* infection over time. The model contains two components: (1) change in the number of infected individuals over time due to transmission, recovery and mass antibiotic treatment with the reported coverage levels, and (2) the observed TF, TI and PCR-positive based on the number of infected individuals. For community *j* (*j* =1, …, 24), we assumed a population of size *N*_*j*_ at the time of treatment *k* (*k* = 1, 2, 3 corresponding to baseline, 12 and 24 months). We used an SIS (susceptible-infectious-susceptible) model structure, assuming that the force of infection is proportional to the prevalence of infection in the population with proportionality constant *β*, and a constant per-capita recovery rate *γ* [11]. Between periods of treatment, we assumed that the probability *p*_*i*,*j*_^(*k*)^(*t*) that there are *i* infections in community *j* at time *t* after treatment time point *k* obeys the following equations [12, 13]:

1 $$ \begin{array}{l}\frac{d{p}_{0,j}^{(k)}}{dt}=\gamma {p}_{1,j}^{(k)}\hfill \\ {}\frac{d{p}_{i,j}^{(k)}}{dt}=\beta \frac{\left(i-1\right)\left({N}_j-i+1\right)}{N_j}{p}_{i-1,j}^{(k)}+\gamma \left(i+1\right){p}_{i+1,j}^{(k)}-\beta \frac{i\left({N}_j-i\right)}{N_j}{p}_{i,j}^{(k)}-\gamma i{p}_{i,j}^{(k)},\kern0.5em \mathrm{f}\mathrm{o}\mathrm{r}\kern0.5em 1\le i\le {N}_j-1\hfill \\ {}\frac{d{p}_{N_j,j}^{(k)}}{dt}=\beta \frac{N_j-1}{N_j}{p}_{N_j-1,j}^{(k)}-\gamma {N}_j{p}_{N_j,j}^{(k)}\hfill \end{array} $$

To model treatment, we assumed that each child aged 0–5 years in community *j* has probability *c*_*j*_^(*k*)^ of receiving treatment with the antibiotic efficacy *e*_*k*_ for treatment period *k*. We modeled each treatment according to $$ {p}_{i,j}^{(k)}\left(t=0\right)={\displaystyle \sum_{i^{\hbox{'}}=i}^{N_j}}{p}_{i^{\hbox{'}},j}^{\left(k,pre\right)}\left(\begin{array}{c}\hfill {i}^{\hbox{'}}\hfill \\ {}\hfill i\hfill \end{array}\right){\left(1-{c}_j^{(k)}\right)}^i{\left({c}_j^{(k)}{e}_k\right)}^{i^{\hbox{'}}-i} $$, where *i* , is the number of infected children aged 0–5 years eligible for treatment, ${p}_{i^{\hbox{'}},j}^{\left(k,pre\right)}$ is the probability of *i* ' infected children aged 0–5 years before treatment time point *k*, and *i* is the number of infected children aged 0–5 years after treatment. Let *S*_*j*,*TF*_^(*l*)^ , *S*_*j*,*TI*_^(*l*)^ and *S*_*j*,*PCR*_^(*l*)^ be the observed TF, TI and PCR-positive at each observation time point *l* (*l* = 0, 1, 2, 3, 4 and 5 corresponding to baseline, 6, 12, 18, 24 and 30 months, respectively) for community *j*. For a community with *i* infections, the probabilities of the observed TF, TI and PCR-positive based on *i* infections are given by using the observation component of the Kalman filter [14] (please see Additional file 1 for more details).

### Sensitivity analyses

We assessed several ways of modeling each observation (TF, and where applicable, TI and PCR). Given a true hidden prevalence (Equation 1), each observation could be specified using epidemiologically driven, traditional sensitivity and specificity, instead of the technically driven, truncated normal distribution described in Additional file 1. With the truncated normal distribution, we have several options to handle the portion of the density at zero, including no zero-inflation, a density at zero (zero-inflation #1) proportional to the density of the normal that would have been <0, or a density at zero given by a free, fitted parameter. In addition, we could include a delay in TF specifically (not TI or PCR), as the follicles associated with TF are known to take months if not longer to recede. Here, we performed specific sensitivity analyses: (a) fitting the model to the observed TF instead of the observed TF, TI and PCR by simplifying Equations 2 and 3 in Additional file 1; (b) using another zero-inflated truncated normal (zero-inflation #2) as the posterior in Equation 2 in Additional file 1 in which the density at 0 % (the first of those 101 discrete units) was assumed to be a parameter between 0 and 1 (*η*_*TF*_ for TF, *η*_*TI*_ for TI, and *η*_*PCR*_ for PCR); (c) using the truncated normal as the posterior; (d) assuming no delay in TF recovery; (e) assuming that the posterior is the convolution of two binomial distributions: the distribution of the number of tested positives *i*_*sens*_ from true positives *i* in a community with *N*_*j*_ individuals because of the sensitivity of a test, and the distribution of the number of tested positives *i*_*spec*_ from true negatives *N*_*j*_ − *i* because of the specificity of the test. Table 1 shows 10 scenarios of sensitivity analyses.

**Table 1:** Forecast scores

Model ID	Log likelihood	Rank	Scenario		
			Density function	Data	Delay in TF recovery
1	−71.864	1	Zero-inflation #1	TF,TI, PCR	Yes
2	−72.207	2	Zero-inflation #1	TF only	Yes
3	−74.792	4	Zero-inflation #2	TF,TI, PCR	Yes
4	−74.576	3	Zero-inflation #2	TF only	Yes
5	−76.640	7	Truncated normal	TF, TI, PCR	Yes
6	−76.782	8	Truncated normal	TF only	Yes
7	−76.173	5	Zero-inflation #1	TF, TI, PCR	No
8	−76.174	6	Zero-inflation #1	TF only	No
9	−122.234	10	Binomial (sensitivity and specificity)	TF, TI, PCR	Yes
10	−105.221	9	Binomial (sensitivity and specificity)	TF only	Yes

### Statistics

Bootstrap percentile confidence intervals for results from Niger were estimated using *R* [15], *N* = 10,000. CIs for model parameter estimates were obtained from MCMC with 16384 (2^14^) steps after a burn-in including 8192 (2^13^) steps.

## Results

Clinical examination (TF and TI) and PCR data were available for 24 communities with 2212 children (aged 0 to 5 years). At the baseline census, communities had a mean of 146 children (95 % CI 137 to 155) aged 0 to 5 years. The mean antibiotic coverage of children was 92.3 % at baseline, 89.0 % at 12 months, and 89.8 % at 24 months. At baseline, the observed TF prevalence in the 24 communities ranged from 10 % to 57 % with a mean prevalence of 27.6 % (95 % CI 21.8 % to 33.9 %). The follow-up TF prevalence was 15.2 % (95 % CI 11.9 % to 18.8 %) at 6 months, 19 % (95 % CI 14.9 % to 23.2 %) at 12 months, 19.7 % (95 % CI 14.6 % to 25.2 %) at 18 months, 14.7 % (95 % CI 10.5 % to 19 %) at 24 months, and 12.3 % (95 % CI 8.5 % to 16.8 %) at 30 months. The observed community prevalence of TF at 36 months which was to be forecasted ranged from 0 % to 30 % with a mean prevalence of 7.6 % (95 % CI 4.9 % to 11.3 %). The observed community prevalence of TF, TI and PCR at each biannual visit are shown in Additional file 2.

**Fig. 1:**
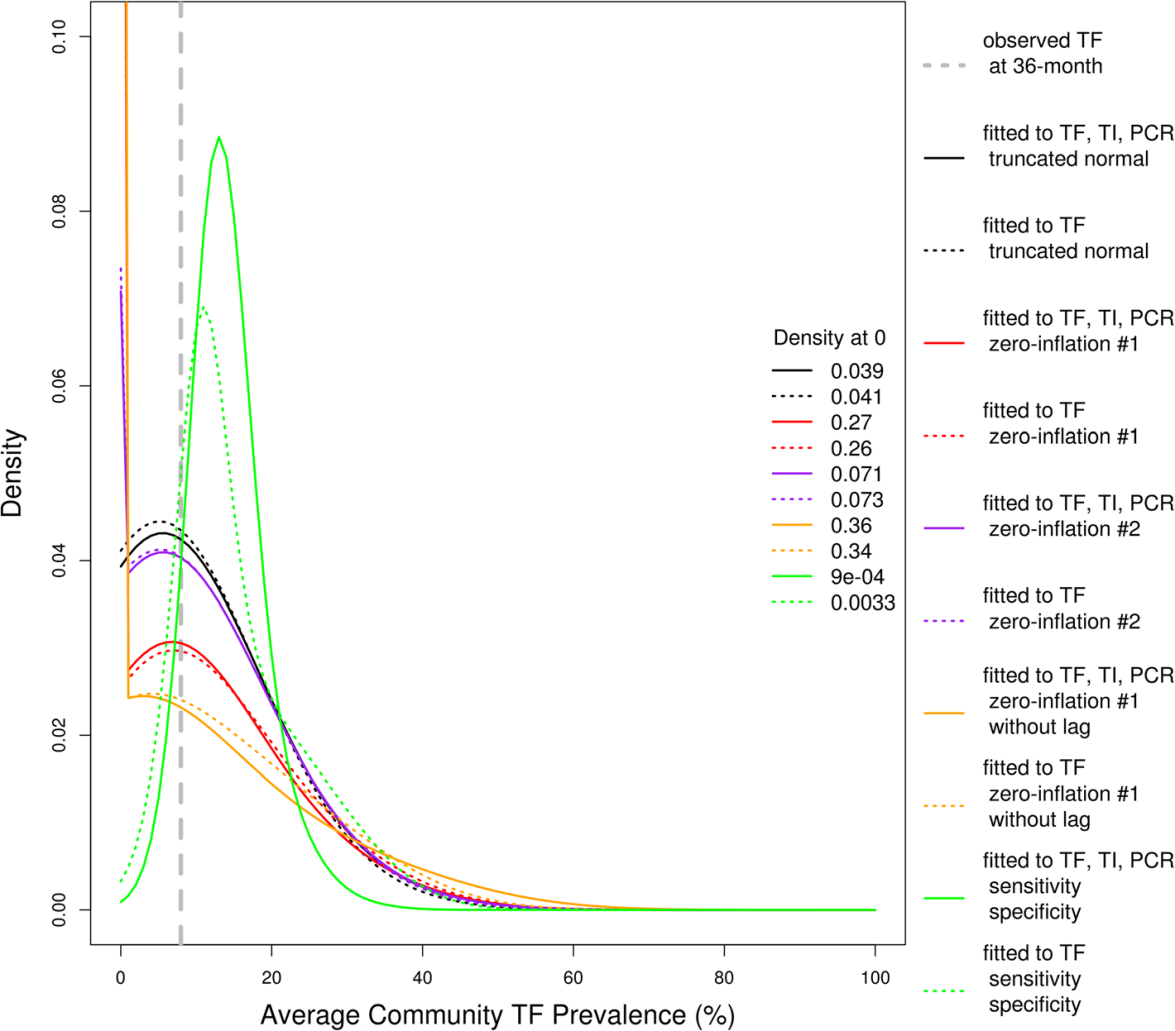
Forecasts of TF prevalence versus observed TF prevalence (averaged over 24 communities). The forecast distributions of average TF prevalence at 36-month in all communities from 10 models are shown by solid and dotted curves in different colors (as listed in the large legends), and their densities at 0 are listed in the small legends. The observed average TF prevalence at 36-month is shown by the dashed grey bar. For the forecast distributions of TF and the observed TF at 36-month in each of 24 communities, please see Additional file 3

**Fig. 2:**
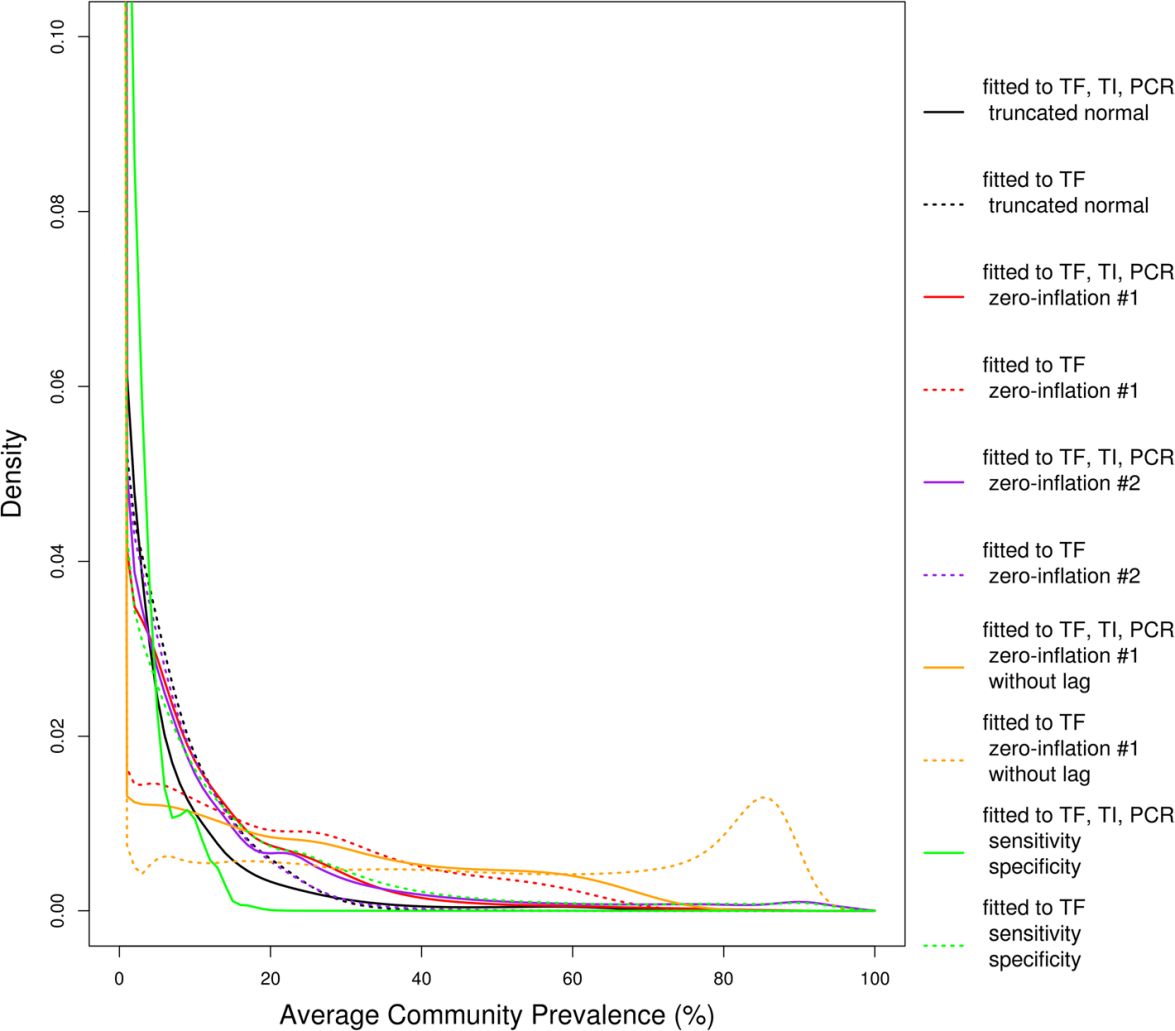
Forecasts of the hidden (true) prevalence (averaged over 24 communities). The forecast distributions of average true prevalence at 36-month in all communities from 10 models are shown by solid and dotted curves in different colors (as listed in legends). For the forecast distributions of true prevalence at 36-month in each of 24 communities, please see Additional file 4

We forecasted distributions of TF prevalence in each of 24 communities from 10 models (Fig. 1 and Additional file 3). The forecast distributions of the hidden (true) prevalence for each of the 10 models (Fig. 2 and Additional file 4) could be markedly different, even if the eventual observation forecasts were similar (Fig. 1 and Additional file 3). The total loglikelihood (the sum of each community’s loglikelihood, Equation 3 in Additional file 1) of the observed 36-month TF prevalence varied between models (Table 1) given the estimated parameters (Table 2). The base case (models 1 and 2: zero-inflation #1 with the observed TF, TI and PCR, and with the observed TF) reveals that the loglikelihood of the observed 36-month TF prevalence from the model based on the observed TF, TI and PCR data from 0 to 30 months was better than the model only based on the observed TF data from 0 to 30 months, although the difference between model 1 and model 2 was not significant (loglikelihood: −71.86 for model 1, −72.21 for model 2, *p =* 0.64, Wilcoxon signed rank test).

**Table 2:** Estimated parameters

Model ID	Estimated parameters^a^ mean (95 % CI)^b^													
	Mean of ***logβ***	SD of ***logβ***	Efficacy of antibiotic	***γ***	***μ***_***TF***_	***σ***_***TF***_	***λ***_***TF***_	***μ***_***TI***_	***σ***_***TI***_	***μ***_***PCR***_	***σ***_***PCR***_	***η***_***TF***_	***η***_***TI***_	***η***_***PCR***_
1	−1.18 (−2.35, −0.782)	0.363 (0.216, 0.754)	0.891 (0.838, 0.944)	0.127 (0.0561, 0.191)	0.816 (0.634, 0.995)	0.245 (0.19, 0.32)	0.571 (0.445, 0.664)	0.166 (0.1, 0.533)	0.0457 (0.0389, 0.0555)	0.316 (0.185, 0.86)	0.0697 (0.0583, 0.0824)	-	-	-
2	−0.57 (−1.31, −0.145)	0.0987 (0.00192, 0.341)	0.888 (0.839, 0.952)	0.231 (0.057, 0.47)	0.657 (0.294, 0.983)	0.231 (0.178, 0.303)	0.55 (0.438, 0.644)	-	-	-	-	-	-	-
3	−1.68 (−2.6, −1.06)	0.779 (0.418, 0.988)	0.937 (0.837, 0.978)	0.08 (0.055, 0.207)	0.358 (0.181, 0.994)	0.296 (0.188, 0.358)	0.591 (0.182, 0.665)	0.135 (0.0618, 0.436)	0.0454 (0.0366, 0.057)	0.24 (0.152, 0.793)	0.0708 (0.058, 0.0849)	0.0225 (0.001, 0.062)	0.33 (0.145, 0.522)	0.182 (0.098, 0.328)
4	−0.922 (−7.48, −0.2)	0.117 (0.006, 0.711)	0.899 (0.838, 0.983)	0.264 (0.061, 0.461)	0.671 (0.107, 0.984)	0.249 (0.166, 0.413)	0.495 (0.302, 0.7)	-	-	-	-	0.0248 (0.001, 0.068)	-	-
5	−1.24 (−2.52, −0.831)	0.629 (0.276, 0.756)	0.928 (0.843, 0.979)	0.352 (0.106, 0.419)	0.862 (0.616, 0.97)	0.295 (0.19, 0.34)	0.597 (0.244, 0.649)	0.272 (0.132, 0.513)	0.0385 (0.0329, 0.0443)	0.596 (0.368, 0.875)	0.0616 (0.0534, 0.0717)	-	-	-
6	−2.84 (−9.56, −0.116)	0.184 (0.00647, 0.634)	0.964 (0.838, 0.995)	0.351 (0.0579, 0.472)	0.702 (0.211, 0.977)	0.308 (0.172, 0.384)	0.592 (0.314, 0.686)	-	-	-	-	-	-	-
7	−0.309 (−1.31, −0.191)	0.161 (0.0695, 0.381)	0.883 (0.838, 0.903)	0.289 (0.156, 0.376)	0.583 (0.508, 0.994)	0.126 (0.112, 0.157)	-	0.0881 (0.0592, 0.364)	0.0471 (0.0373, 0.0583)	0.183 (0.132, 0.609)	0.0703 (0.0583, 0.0844)	-	-	-
8	−0.123 (−0.637, −0.0199)	0.0411 (0.001, 0.164)	0.859 (0.837, 0.896)	0.0995 (0.055, 0.271)	0.301 (0.251, 0.814)	0.121 (0.107, 0.141)	-	-	-	-	-	-	-	-
9	−3.73 (−9.74, −2.45)	0.774 (0.0932, 0.995)	0.886 (0.857, 0.911)	0.16 (0.149, 0.166)	0.652 (0.612, 0.823)	0.897 (0.879, 0.907)	0.315 (0.247, 0.356)	0.58 (0.509, 0.783)	0.984 (0.98, 0.986)	0.627 (0.575, 0.887)	0.963 (0.957, 0.966)	-	-	-
10	−1.53 (−7.76, −1.16)	0.651 (0.0369, 0.953)	0.966 (0.855, 0.998)	0.0833 (0.0572, 0.165)	0.536 (0.501, 0.904)	0.903 (0.887, 0.916)	0.242 (0.18, 0.348)	-	-	-	-	-	-	-

^a^: please see Additional file 1 for the interpretations of parameters; for the observation model with sensitivity and specificity (models 9 and 10), *μ*_*TF*_ is the sensitivity of TF, *σ*_*TF*_ is the specificity of TF, *μ*_*TI*_ is the sensitivity of TI, *σ*_*TI*_ is the specificity of TI, *μ*_*PCR*_ is the sensitivity of PCR, and *σ*_*PCR*_ is the specificity of PCR

^b^: 95 % CI was obtained from MCMC with 16384 steps after a burn-in including 8192 steps

As a sensitivity analysis, we assumed no delay in TF recovery (models 7 and 8) and compared the model without the delay in TF recovery to the base case (models 1 and 2), and found that the model with the delay in TF recovery and TF, TI and PCR data was significantly better than the model with the TF data and no delay in TF recovery (loglikelihood: −71.86 for model 1, −76.17 for model 8, *p* = 0.003), and the model with the delay in TF recovery had better likelihood than the model without the delay in TF recovery for both scenarios: multiple observations of TF, TI and PCR (loglikelihood: −71.86 for model 1, −76.17 for model 7, *p* = 0.002), and the observation of TF only (loglikelihood: −72.21 for model 2, −76.17 for model 8, *p* = 0.005). Sensitivity analyses found no significant differences between model 3 and model 4 (loglikelihood: −74.79 for model 3, −74.58 for model 4, *p* = 0.60), model 5 and model 6 (loglikelihood: −76.64 for model 5, −76.78 for model 6, *p* = 0.35), and model 7 and model 8 (loglikelihood: −76.173 for model 5, −76.174 for model 6, *p* = 0.16), except for model 9 and model 10 (loglikelihood: −122.23 for model 9, −105.22 for model 10, *p* = 0.01). Sensitivity analyses on models based on TF, TI and PCR data but with different posterior densities (models 1, 3, 5 and 9) show that the zero-inflation #1 (model 1) was better than other models, and the observation model with the sensitivity and specificity did not improve forecasts and had the lowest likelihood (Table 1).

Models 9 and 10 allowed estimation of sensitivity and specificity for each of TF, TI and PCR (Table 3). TF was estimated to be 65.2 % (95 % CI 61.2 % to 82.3 %) sensitive and 89.7 % (95 % CI 87.9 % to 90.7 %) specific; the delay in TF recovery was estimated to be 0.32 per six-month (95 % CI 0.25 to 0.36); TI was estimated to be 58 % (95 % CI 50.9 % to 78.3 %) sensitive and 98.4 % (95 % CI 98 % to 98.6 %) specific, and PCR was estimated to be 62.7 % (95 % CI 57.5 % to 88.7 %) sensitive and 96.3 % (95 % CI 95.7 % to 96.6 %) specific.

**Table 3:** Estimated sensitivity and specificity

Test	Sensitivity	Specificity	Delay in TF recovery (per six-month)
	Mean (95 % CI)^a^	Mean (95 % CI)^a^	Mean (95 % CI)^a^
Model 9			
TF	0.65 (0.61, 0.82)	0.90 (0.88, 0.91)	0.32 (0.25, 0.36)
TI	0.58 (0.51, 0.78)	0.98 (0.98, 0.99)	-
PCR	0.63 (0.58, 0.89)	0.96 (0.96, 0.97)	-
Model 10			
TF	0.54 (0.50, 0.90)	0.90 (0.89, 0.92)	0.24 (0.18, 0.35)

^a^: 95 % CI was obtained from MCMC with 16384 steps after a burn-in including 8192 steps

## Discussion and conclusion

Here, we compared the inclusion of several features of model-based forecasts of the prevalence of clinically active trachoma (TF) 6-months into the future. Inclusion of the second trachoma sign TI and lab-based chlamydia testing improved TF forecasts, but not significantly. Thus we cannot state that PCR testing is necessary for better forecasting of TF, although it may well help forecast future infection—that would require further study. TF is known to remain for months after infection has cleared, and inclusion of this feature into the observation model improved forecasting significantly. Different forms of the observation portion of the hidden model performed differently. In particular, a zero-inflated truncated normal distribution performed better than the observation model with sensitivity and specificity, and inclusion of the sensitivity and specificity into the observation model did not improve forecasts.

We estimated the sensitivity and specificity for each of TF, TI and PCR. Our estimates of TF sensitivity (65.2 % from 61.2 % to 82.3 %) and specificity (89.7 % from 87.9 % to 90.7 %) are consistent with the estimates in studies [5, 16–18] in Tanzania and The Gambia, in which sensitivity was estimated at 24.0 % to 86.7 % and specificity at 74.0 % to 94.0 %. However, in study [3] in Ethiopia, TF sensitivity was estimated to be 87.3 % (higher than our estimate of TF sensitivity), and TF specificity was estimated to be 36.6 % (lower than our estimate of TF specificity). Our estimates of TI sensitivity (58.0 % from 50.9 % to 78.3 %) and specificity (98.4 % from 98.0 % to 98.6 %) closely agree with the estimates in [3, 5, 16, 17] in which sensitivity was estimated at 12.4 % to 77.0 % and specificity at 74.0 % to 99.4 %. The estimate of PCR specificity (96.3 % from 95.7 % to 96.6 %) in our study is close to the estimates in [3, 5, 17] in which PCR specificity was estimated at 93.0 % to 100 %. However, our estimate of PCR sensitivity (62.7 % from 57.5 % to 88.7 %) is lower than the estimates in [3, 5, 17] in which PCR sensitivity was estimated at 77.8 % to 97.0 %.

Previous mathematical models have provided insight into the transmission of trachoma [3, 5, 16, 17, 19–24], evaluated the sensitivity and specificity of diagnostic tests for ocular chlamydia infection including the clinical signs of TF and TI and PCR-based assay in the absence of a gold standard [3, 5, 25]. Recently, a regression model was used to forecast the TF prevalence after a number of years of mass drug administration given the ITI database [26]. A hidden Markov model was used to estimate the sensitivity and specificity of TF test based on the data collected from East and West Africa [5]. A latent class analysis was performed to estimate the sensitivity and specificity of TF test based on the data from randomly selected 40 villages in Ethiopia (the Trachoma Elimination Follow-up study) [3]. In [26], linear and logistic regression modeling was applied to a comprehensive database of trachoma prevalence to investigate the effect of MDA on baseline TF prevalence. In our previous study [27], we compared the forecasts of trachoma prevalence by expert opinion, statistical regression and transmission models using the data from 24 villages in Niger (PRET study). However, the delay in TF recovery was not included in [3, 5, 26] and [27]; [3] and [26] did not use the process model; the forecasts made by [27] were only based on the observed PCR data.

Here, we forecasted trachoma in the short term of 6 months. Longer term models would be of more practical use to control programs and can be studied in the future. The process model used in this study did not include strain diversity [28], household-level risk factors [29], and infection from outside the population of children aged 0–5 years in each community [23]. Models could be further refined to reflect age-structured transmission. In this setting, the older children and adults were being treated as well, and other studies have shown consistently higher prevalence in small children than in other age groups (e.g. [30, 31]). The use of results from 24 communities allowed us to separate the performance of several models—larger studies should be able to discriminate smaller differences. It should be noted that here, due to the available study data, we studied 0–5 year-old children, whereas the WHO criteria are defined in terms of 1–9 year-old children. Communities were geographically separated villages, although we cannot rule out transmission between villages.

In this study, we used a transmission model to forecast TF prevalence. The results demonstrated that using the delay in TF recovery in the model could improve the forecasts of TF prevalence. Because a recovery period may follow the clearance of infection and the follicles so characteristic of active trachoma may linger for weeks, in which case the clinical exam would falsely indicate the presence of *Chlamydia trachomatis* [32]. We did not find a significant difference between the forecasts of TF prevalence based on the observed TF data and based on the observed TF, TI and PCR data. Current WHO guidelines for starting MDA are based on the district prevalence of TF in children (aged 1–9 years), and the results in this study (based on children aged 0–5 years) suggest that future studies could consider use of the delay in TF recovery and TF data to assess forecasting at district level.

In many districts worldwide, the assessment of 2020 goals will likely include estimation of TF prevalence from surveys that had been done earlier than 2020. These estimations could take into account the features found here to be useful in forecasting. It is also possible that as we near elimination, a laboratory indication of infection status supplants clinical signs such as TF—forecasting of a laboratory sign of infection might require a different analysis than that presented here.

## Additional files

Additional file 1:
**Modeling methods.** (PDF 336 kb) Additional file 2:
**Observed TF, TI and PCR prevalence.** (PDF 11 kb) Additional file 3:
**Forecasts of TF prevalence versus observed TF prevalence.** The forecast distributions of TF prevalence at 36-month in each of 24 communities from 10 models are shown by solid and dotted curves in different colors (as listed in the large legends), and their densities at 0 in each community are listed in the small legends. The observed TF prevalence in each community at 36-month is shown by the dashed grey bar. (TIFF 2817 kb) Additional file 4:
**Forecasts of the hidden (true) prevalence.** The forecast distributions of true prevalence at 36-month in each of 24 communities from 10 models are shown by solid and dotted curves in different colors (as listed in legends). (TIFF 1931 kb)
