# Erratum to: Short-term forecasting of the prevalence of clinical trachoma: utility of including delayed recovery and tests for infection

**DOI:** 10.1186/s13071-016-1588-0

**Published:** 2016-06-09

**Authors:** Fengchen Liu, Travis C. Porco, Abdou Amza, Boubacar Kadri, Baido Nassirou, Sheila K. West, Robin L. Bailey, Jeremy D. Keenan, Thomas M. Lietman

**Affiliations:** F.I. Proctor Foundation, University of California San Francisco, 513 Parnassus, Medical Sciences 309A, San Francisco, CA 94143-0944 USA; Department of Ophthalmology, University of California San Francisco, San Francisco, CA USA; Department of Epidemiology and Biostatistics, University of California San Francisco, San Francisco, CA USA; Programme FSS/Université Abdou Moumouni de Niamey, Programme National de Santé Oculaire, Niamey, Niger; Dana Center for Preventive Ophthalmology, Wilmer Eye Institute, Johns Hopkins University, Baltimore, MD USA; Clinical Research Unit, Department of Infectious and Tropical Diseases, London School of Hygiene and Tropical Medicine, London, UK

## Erratum

After publication of the original article [1], the authors noticed an error in the Acknowledgements section. One of the grant numbers listed as U01GM08778 should instead be U01-GM087728. The correct version of the Acknowledgements section is included below.

### Acknowledgements

In addition to the study sponsors the Bill and Melinda Gates Foundation (grant number 48027) and MIDAS (grant number U01-GM087728), the authors gratefully acknowledge funding of the NTD Modelling Consortium by the Bill and Melinda Gates Foundation in partnership with the Task Force for Global Health. The views, opinions, assumptions or any other information set out in this article are solely those of the authors. The authors thank the data and safety monitoring committee, including Douglas Jabs, MD, MBA (chair), Antoinette Darville, MD, Maureen Maguire, PhD, and Grace Saguti, MD, who were generous with their time and advice and met before and during the study. The authors thank all of our colleagues in Niger at Programme National de Santé Oculaire who collected samples and data. This trial is registered at ClinicalTrials.gov, number NCT00792922. We also thank the NTD support center and the Task Force for Global Health.
