# Supplementary material for: Short-term forecasting of the prevalence of clinical trachoma: utility of including delayed recovery and tests for infection
Source: Parasit Vectors. 2015 Oct 22;8:535. doi: 10.1186/s13071-015-1115-8 (PMC4618840; doi:10.1186/s13071-015-1115-8)

Additional file 1.

Modeling methods

We constructed a stochastic transmission model of *Chlamydia trachomatis* infection over time. The model contains two components: (1) change in the number of infected individuals over time due to transmission, recovery and mass antibiotic treatment with the reported coverage levels, and (2) the observed TF, TI and PCR-positive based on the number of infected individuals (as shown in Figure 1). For community j (j =1, …, 24), we assumed a population of size N_j at the time of treatment k (k = 1, 2, 3 corresponding to baseline, 12 and 24 months). We used an SIS (susceptible-infectious-susceptible) model structure, assuming that the force of infection is proportional to the prevalence of infection in the population with proportionality constant β, and a constant per-capita recovery rate γ [1]. Between periods of treatment, we assumed that the probability $p_{i,j}^{(k)}(t)$ that there are i infections in community j at time t after treatment time point k obeys the following equations [2, 3]:

$$\frac{dp_{0,j}^{(k)}}{dt} = \gamma p_{1,j}^{(k)}$$

$$\frac{dp_{i,j}^{(k)}}{dt} = \beta \frac{(i-1)(N_j-i+1)}{N_j} p_{i-1,j}^{(k)} + \gamma(i+1)p_{i+1,j}^{(k)} - \beta\frac{i(N_j-i)}{N_j}p_{i,j}^{(k)} - \gamma i p_{i,j}^{(k)}, \text{ for } 1 \leq i \leq N_j - 1 \qquad (1)$$

$$\frac{dp_{N_j,j}^{(k)}}{dt} = \beta\frac{N_j-1}{N_j}p_{N_j-1,j}^{(k)} - \gamma N_j p_{N_j,j}^{(k)}$$

To model treatment, we assumed that each child aged 0-5 years in community j has probability $c_j^{(k)}$ of receiving treatment with the antibiotic efficacy e_k for treatment period k. We modeled each treatment according to $p_{i,j}^{(k)}(t=0) = \sum_{i'=i}^{N_j} p_{i',j}^{(k,pre)} \binom{i'}{i} \left(1-c_j^{(k)}\right)^{i} \left(c_j^{(k)}e_k\right)^{i'-i}$, where i' is the number of infected children aged 0-5 years eligible for treatment, $p_{i',j}^{(k,pre)}$ is the probability of i' infected children aged 0-5 years before treatment time point k, and i is the number of infected children aged 0-5 years after treatment. Let $S_{j,TF}^{(l)}$, $S_{j,TI}^{(l)}$ and $S_{j,PCR}^{(l)}$ be the observed TF, TI and PCR-positive at each observation time point l (l = 0, 1, 2, 3, 4 and 5 corresponding to baseline, 6, 12, 18, 24 and 30 months, respectively) for community j. From community j with population size N_j of which the number of infections Y_j equals i, the probabilities that s of TF, TI or PCR-positive are observed from i infections are given by using the observation component of the Kalman filter assuming that the posterior density of the observation given the number of infections is Gaussian [4]:

$$P(S_{j,TF} = s | Y_j = i) = \mathcal{N}\left(s; \lambda_{TF} S_{j,TF'} + (1-\lambda_{TF}) i\mu_{TF}, \left((1-\lambda_{TF})\sigma_{TF}\right)^2\right)$$
$$P(S_{j,TI} = s | Y_j = i) = \mathcal{N}(s; i\mu_{TI}, \sigma_{TI}^2) \quad (2)$$
$$P(S_{j,PCR} = s | Y_j = i) = \mathcal{N}(s; i\mu_{PCR}, \sigma_{PCR}^2)$$

where $\mathcal{N}(x; \theta_1, \theta_2)$ is a Gaussian density with argument x, mean θ_1, and covariance θ_2; λ_{TF} is the autocorrelation between the observed TF at the previous and current time points (delay in TF recovery); $S_{j,TF'}$ is the observed TF at the previous time point; μ_{TF}, μ_{TI} and μ_{PCR} are correlations between the number of infections in community j and its observed TF, TI and PCR-positive; σ_{TF}^2, σ_{TI}^2 and σ_{PCR}^2 are correlations between the number of infections in community j and its observed TF, TI and PCR-positive. We assumed a standard beta-binomial prior $P(Y_j = i) = \binom{N_j}{i} \frac{\mathrm{B}(i+\alpha, N_j - i + \rho)}{\mathrm{B}(\alpha, \rho)}$ (where the shape parameters α and ρ for each treatment were computed from the observed distribution of infection of 24 communities at baseline, 12 and 24 months, $\mathrm{B}(z_1, z_2)$ is the beta function [5]) as the distribution of pre-treatment prevalence $p_{i,j}^{(k,pre)}$. Given i infections in community j, we computed the probability of the observed TF-positives of treatment k in community j according to $P\left(S_{j,TF} = S_{j,TF}^{(l)}\right) = \sum_{i=0}^{N_j} p_{i,j}^{(k)}(\tau) P\left(S_{j,TF} = S_{j,TF}^{(l)} \middle| Y_j = i\right)$ (where $S_{j,TF}^{(l)}$ denotes the observed TF-positive at one of the observation time points in the period k; τ (6 or 12 months) is the interval between treatment and observation time points; l ($l = 2k - 1$ for $\tau = 6$, and $l = 2k$ for $\tau = 6$) is the observation time point). Similarly, the probabilities of the observed TI and PCR-positive of treatment k in community j were computed according to $P\left(S_{j,TI} = S_{j,TI}^{(l)}\right) = \sum_{i=0}^{N_j} p_{i,j}^{(k)}(\tau) P\left(S_{j,TI} = S_{j,TI}^{(l)} \middle| Y_j = i\right)$, and $P\left(S_{j,PCR} = S_{j,PCR}^{(l)}\right) = \sum_{i=0}^{N_j} p_{i,j}^{(k)}(\tau) P\left(S_{j,PCR} = S_{j,PCR}^{(l)} \middle| Y_j = i\right)$, respectively. We assumed independent communities, so that the total loglikelihood of the observed TF, TI and PCR-positive at τ months after each treatment k may be computed by summing over all 24 communities:

$$\sum_{j=1}^{24}\left(\log\left(\sum_{i=0}^{N_j} p_{i,j}^{(k)}(\tau) P\left(S_{j,TF} = S_{j,TF}^{(l)} \middle| Y_j = i\right)\right) + \log\left(\sum_{i=0}^{N_j} p_{i,j}^{(k)}(\tau) P\left(S_{j,TI} = S_{j,TI}^{(l)} \middle| Y_j = i\right)\right) + \log\left(\sum_{i=0}^{N_j} p_{i,j}^{(k)}(\tau) P\left(S_{j,PCR} = S_{j,PCR}^{(l)} \middle| Y_j = i\right)\right)\right) \quad (3)$$

For simplicity, we assumed a constant population (children aged 0-5 years) size for all communities (N_j=100). The parameters in the model were optimized by using the Metropolis algorithm with the total likelihood of three treatment periods to fit the model to the observed TF, TI and PCR-positive in each community at 6, 12, 18, 24 and 30 months [6]. In optimization, the likelihood based on Equation 2 was obtained by using a zero-inflated truncated normal

distribution (zero-inflation #1, which is similar to a truncated normal between 0% and 100%, but the density at 0% was assumed to be the integral from negative infinity to 0 from the normal distribution), that is, the posterior was assumed be the zero-inflated truncated normal with 101 discrete units between 0 and 1. Given the observed TF, TI and PCR-positive in each community at 6, 12, 18, 24 and 30 months, the distribution of the observed TF-positive (101 discrete units corresponding to 0%, 1%, …, 100% of prevalence) in a community at 36 months was forecasted by using a Hidden Markov model (specifically, a Markov-switching AR(1) model [7] with the delay in TF recovery, because the observed TF at the previous and current time points are not conditionally independent) according to the equation of forecast distributions [7] and the observation component of the Kalman filter in Equation 2.

References

1. Brauer F, van den Driessche P, Wu J: **Mathematical Epidemiology**. Berlin: Springer-Verlag; 2008.
2. Ray KJ, Lietman TM, Porco TC, Keenan JD, Bailey RL, Solomon AW, Burton MJ, Harding-Esch E, Holland MJ, Mabey D: **When can antibiotic treatments for trachoma be discontinued? Graduating communities in three african countries**. *PLoS neglected tropical diseases* 2009, **3**(6):e458.
3. Lietman TM, Gebre T, Ayele B, Ray KJ, Maher MC, See CW, Emerson PM, Porco TC, Group TS: **The epidemiological dynamics of infectious trachoma may facilitate elimination**. *Epidemics* 2011, **3**(2):119-124.
4. Ristic B, Arulampalam S, Gordon N: **Beyond the Kalman filter**. *IEEE AEROSPACE AND ELECTRONIC SYSTEMS MAGAZINE* 2004, **19**(7):37-38.
5. Johnson NL, Kotz S, Kepm AW: **Univariate Discrete Distributions**. New York: John Wiley & Sons, Inc; 1993.
6. Brooks S, Gelman A, Jones GL, Meng XL: **Handbook of Markov Chain Monte Carlo Preface**. *Ch Crc Handb Mod Sta* 2011:Xix-Xx.
7. Zucchini W, MacDonald IL: **Hidden Markov models for time series : an introduction using R**. Boca Raton: CRC Press; 2009.

Figure 1. Model Structure

The process model (the true hidden prevalence, TP, at six observation time points (l = 0, 1, 2, 3, 4 and 5)) is shown by red circles and arrows. The observation models (the observed TF, TI and PCR at observation time points) are shown by blue (TF) and green (TI and PCR) circles, which are based on the true hidden prevalence (indicated by black arrows). The delay in TF recovery in the TF observation model is shown by blue arrows.

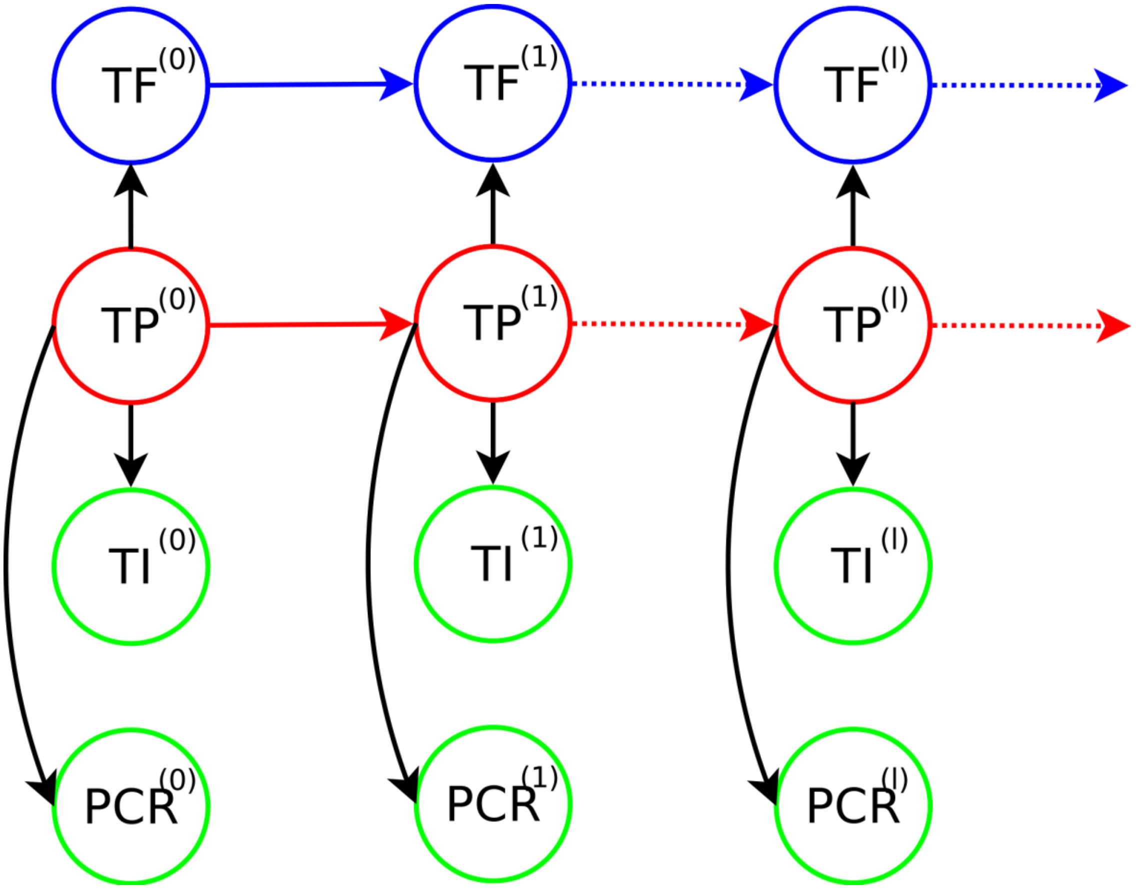

Supplement: Additional file 1: — Modeling methods. (PDF 336 kb) [file 13071_2015_1115_MOESM1_ESM.pdf]
