# Supplementary material for: Short-term forecasting of the prevalence of clinical trachoma: utility of including delayed recovery and tests for infection
Source: Parasit Vectors. 2015 Oct 22;8:535. doi: 10.1186/s13071-015-1115-8 (PMC4618840; doi:10.1186/s13071-015-1115-8)
Supplement: Additional file 2: — Observed TF, TI and PCR prevalence. (PDF 11 kb) [file 13071_2015_1115_MOESM2_ESM.pdf]

Additional file 2. Observed TF, TI and PCR prevalence

Antibiotic coverage			Observed prevalence of TI in children 0-5 years							Observed prevalence of PCR in children 0-5 years							Observed prevalence of TF in children 0-5 years						
**0**	**12**	**24**	**0**	**6**	**12**	**18**	**24**	**30**	**36**	**0**	**6**	**12**	**18**	**24**	**30**	**36**	**0**	**6**	**12**	**18**	**24**	**30**	**36**
81%	81%	86%	14%	5%	10%	6%	6%	0%	0%	30%	3%	3%	5%	6%	3%	12%	48%	17%	27%	32%	10%	14%	**14%**
83%	89%	92%	2%	0%	2%	0%	0%	0%	0%	8%	2%	2%	0%	0%	0%	0%	10%	21%	9%	4%	5%	0%	**10%**
91%	79%	89%	1%	2%	12%	4%	0%	0%	4%	8%	0%	0%	1%	4%	2%	2%	14%	4%	14%	14%	9%	9%	**9%**
89%	91%	90%	21%	2%	5%	8%	2%	0%	15%	51%	20%	14%	0%	0%	5%	23%	47%	29%	38%	29%	27%	50%	**28%**
87%	86%	80%	7%	0%	12%	1%	1%	0%	0%	25%	3%	3%	1%	3%	0%	0%	17%	13%	11%	4%	11%	5%	**3%**
91%	90%	92%	5%	5%	3%	3%	0%	0%	0%	13%	4%	2%	0%	0%	0%	0%	41%	15%	16%	20%	5%	13%	**1%**
92%	88%	89%	2%	0%	0%	0%	0%	0%	0%	2%	0%	0%	0%	0%	0%	0%	11%	3%	0%	0%	0%	0%	**0%**
88%	84%	83%	6%	0%	0%	0%	0%	0%	0%	20%	0%	3%	3%	3%	0%	0%	20%	4%	8%	5%	3%	3%	**0%**
89%	81%	85%	11%	2%	2%	5%	0%	0%	1%	3%	1%	1%	1%	1%	0%	0%	11%	9%	12%	10%	1%	14%	**4%**
96%	84%	85%	17%	2%	7%	1%	2%	0%	2%	28%	4%	6%	3%	2%	0%	2%	38%	20%	27%	33%	25%	8%	**9%**
87%	88%	85%	6%	0%	0%	0%	0%	0%	4%	7%	4%	6%	4%	2%	0%	0%	11%	5%	13%	14%	12%	7%	**2%**
92%	87%	91%	14%	0%	3%	5%	4%	1%	1%	48%	0%	0%	9%	25%	8%	16%	56%	18%	23%	57%	10%	19%	**4%**
95%	82%	90%	1%	0%	1%	0%	0%	3%	0%	3%	1%	3%	0%	1%	3%	5%	14%	4%	1%	9%	7%	13%	**0%**
96%	92%	90%	7%	3%	3%	2%	3%	5%	0%	39%	11%	13%	3%	5%	5%	8%	32%	24%	15%	23%	22%	16%	**10%**
99%	92%	91%	9%	6%	3%	6%	20%	6%	0%	31%	2%	6%	28%	28%	19%	11%	22%	32%	25%	37%	18%	27%	**11%**
97%	96%	94%	22%	2%	19%	13%	1%	7%	1%	35%	9%	20%	14%	15%	18%	22%	51%	22%	24%	17%	26%	21%	**30%**
100%	95%	80%	3%	3%	4%	2%	0%	3%	0%	9%	5%	4%	7%	13%	6%	13%	24%	16%	16%	9%	37%	12%	**0%**
98%	94%	95%	26%	2%	2%	3%	0%	2%	1%	58%	9%	11%	1%	2%	0%	0%	57%	32%	33%	32%	34%	13%	**13%**
96%	97%	97%	2%	0%	3%	5%	1%	0%	0%	27%	10%	7%	1%	5%	0%	0%	20%	6%	17%	9%	14%	2%	**0%**
90%	97%	95%	3%	1%	8%	9%	2%	2%	2%	24%	6%	16%	10%	18%	8%	5%	25%	17%	42%	30%	29%	12%	**12%**
95%	94%	93%	1%	3%	0%	0%	0%	0%	0%	11%	2%	2%	0%	0%	0%	0%	14%	10%	13%	13%	3%	1%	**1%**
97%	95%	92%	14%	0%	3%	2%	0%	0%	12%	17%	3%	1%	1%	5%	10%	13%	23%	16%	28%	20%	21%	13%	**10%**
91%	94%	97%	2%	1%	5%	0%	0%	0%	0%	5%	2%	1%	1%	0%	1%	0%	19%	10%	17%	19%	4%	15%	**4%**
96%	82%	94%	6%	4%	0%	7%	0%	0%	5%	6%	14%	4%	7%	2%	2%	7%	38%	20%	27%	33%	19%	7%	**14%**
