# Supplementary figures and images for: Short-term forecasting of the prevalence of clinical trachoma: utility of including delayed recovery and tests for infection
Source: Parasit Vectors. 2015 Oct 22;8:535. doi: 10.1186/s13071-015-1115-8 (PMC4618840; doi:10.1186/s13071-015-1115-8)

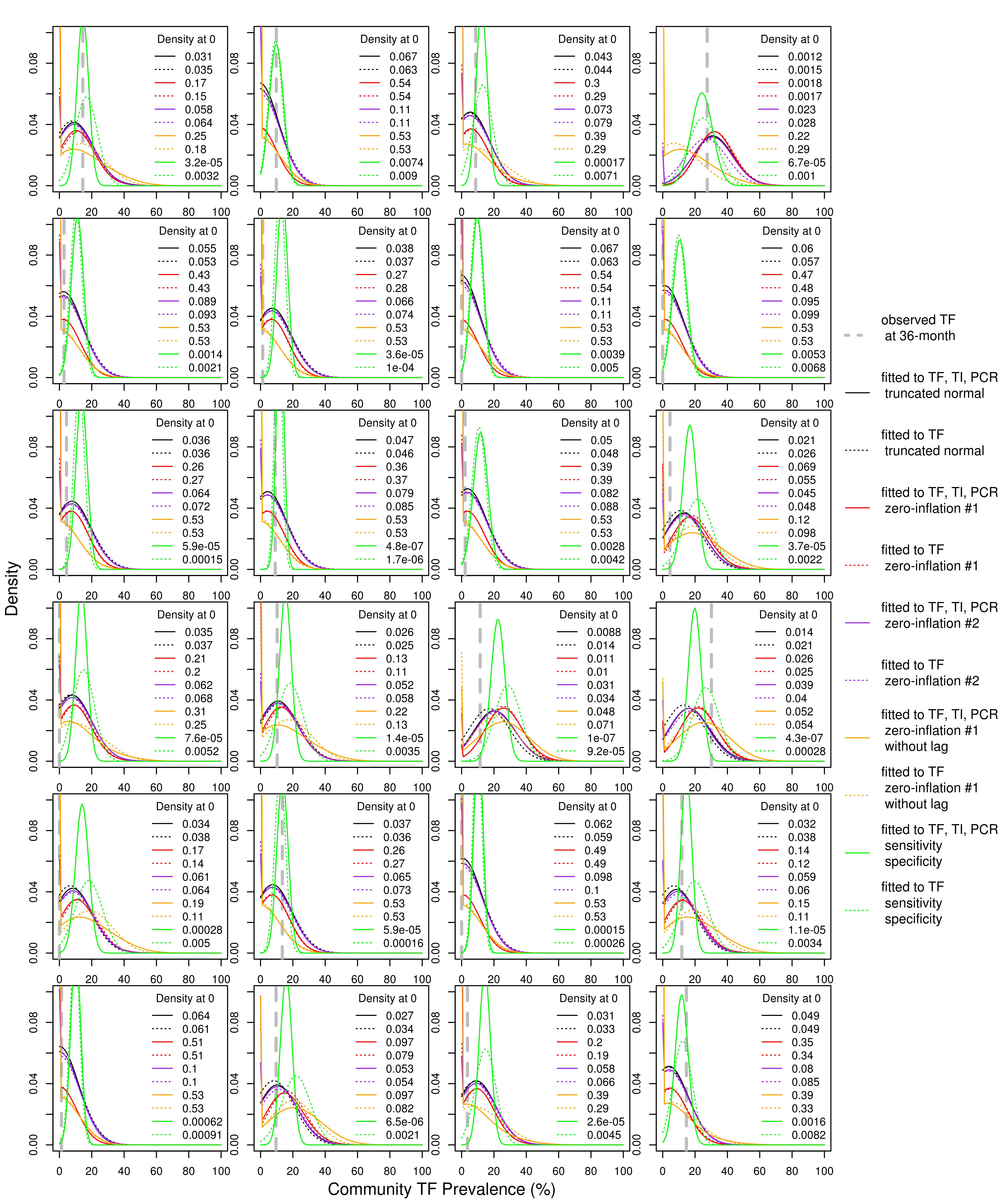

Supplement: Additional file 3: — Forecasts of TF prevalence versus observed TF prevalence. The forecast distributions of TF prevalence at 36-month in each of 24 communities from 10 models are shown by solid and dotted curves in different colors (as listed in the large legends), and their densities at 0 in each community are listed in the small legends. The observed TF prevalence in each community at 36-month is shown by the dashed grey bar. (TIFF 2817 kb) [file 13071_2015_1115_MOESM3_ESM.tiff]

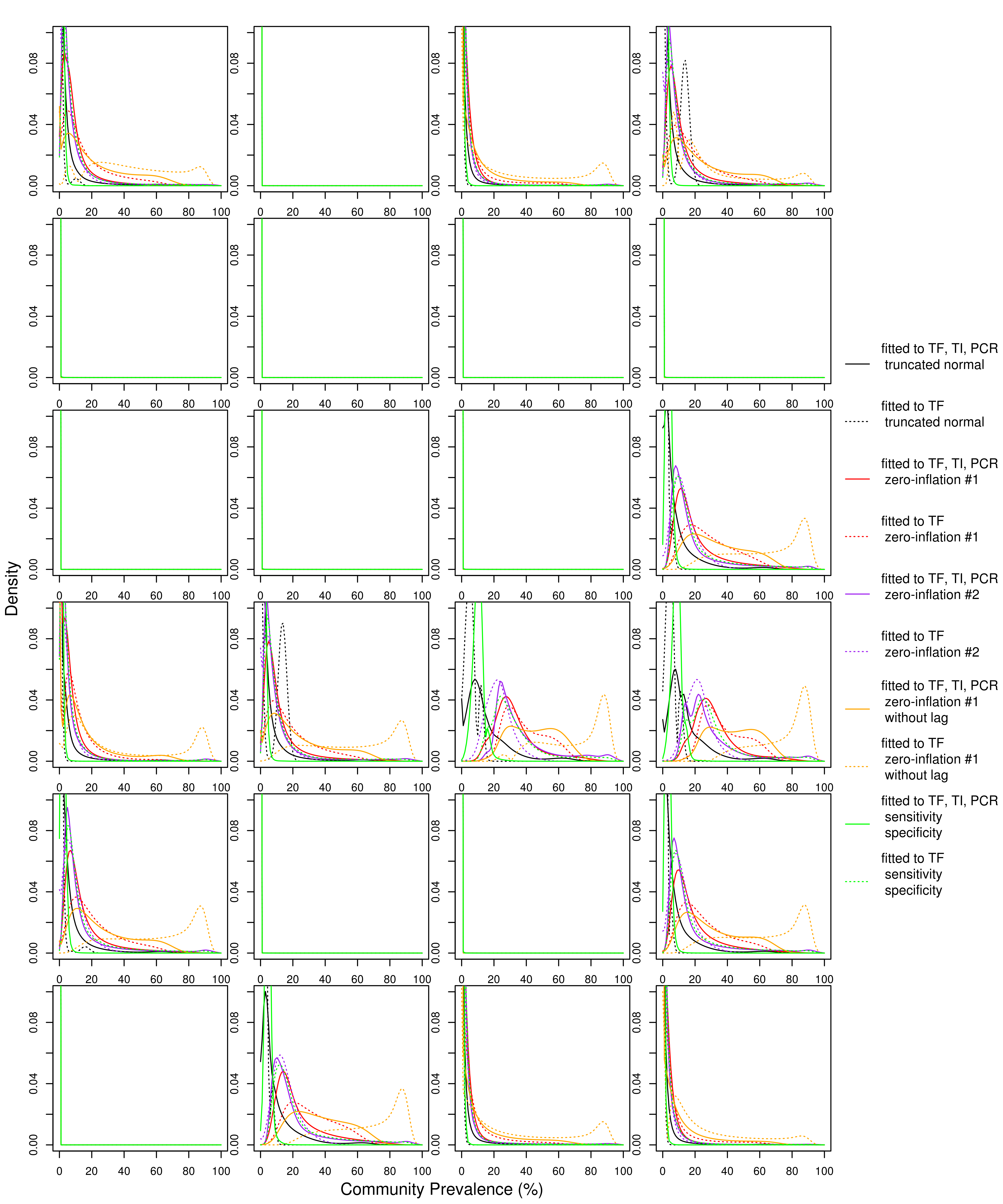

Supplement: Additional file 4: — Forecasts of the hidden (true) prevalence. The forecast distributions of true prevalence at 36-month in each of 24 communities from 10 models are shown by solid and dotted curves in different colors (as listed in legends). (TIFF 1931 kb) [file 13071_2015_1115_MOESM4_ESM.tiff]
